# Our Correspondence

**Published:** 1871-07

**Authors:** 


### Our Correspondence.

AN OLD SWINDLER UNDER NEW COLORS.—An invalid sends us the following precious documents, which we publish for the benefit of those who are disposed to patronize every abominable advertising humbug that comes in their way.—Our correspondent seeing the following benevolent notice in his village paper, bit at once, and wrote the kind old chap for his *free* advice. This is the notice:

MURDER! BEWARE OF QUACKS.—50,000, both sexes, hurried to premature graves yearly, through nervous debility, &c., produced by youthful indiscretion. The advertiser, during years of suffering, tried every advertised remedy without relief; has discovered a simple means of self-cure, which he will send *free* to his fellow sufferers.
Address, S. R. SHAW,
737 Broadway, New York.

In obedience to which he wrote and received the following exceedingly charitable document in return. Read it, and then bless your stars that such self-sacrificing specimens of humanity can be found in our land, to soothe the lingering sufferer to the grave:

737 Broadway,
New York, 1871.

DEAR FRIEND:

Yours is at hand. Enclosed you will find descriptive pamphlet of your disease. Read it carefully and decide, and if afflicted enclose me the price of Dr. Young's Mechanical Cure and Vigor Pills. I spent over $600 with various quacks and imposters, including J. H. Tuttle, James Butler, E. Traver, J. B. Ogden, Drs. La Croix, Hunter, Brown, McLain, DeLos, Kline, Howard Association. I also spent $30 for Buchu, and all failed to give me any relief. When nearly at the point of death, I was told by a physician to try Dr. Young's Cure and pills. I paid him ($5) for them. I began to rapidly recover and gained 38 pounds of flesh in three months. Being desirous to aid my fellow sufferers, I have made arrangements with the doctor to furnish his Cure and Pills to all who need it at exactly cost price, ($5). My conscience of doing good being ample reward for my time and trouble. And now, my afflicted friend, if you do not feel able to spare that amount at once, you may send me $3, and I will forward the remedies, and you can send me the balance ($2) when cured. Do not despair, you can be cured. Your Sympathising Friend,

S. R. SHAW.

P. S.—I feel so positive of curing you, that if I fail after six weeks' trial, you can enclose me the *Instrument* by mail and I will cheerfully return your money by return mail. Some evil disposed person has copied my Circular for pecuniary benefit and is imposing upon the public thereby; of such I would say—Beware.

S. R. S.

Now, did you ever read a more sympathising letter than that? Why, that chap has spent hundreds of dollars for you; has gone to a great expense in having the above letter lithographed, so as to resemble a real, *bona fide*, written letter. He has also advertised in all the country newspapers, for which he pays liberally, and now offers you "Dr. Young's Cure and Pills" "*at exactly cost price!*" Only think of it! We declare, such benevolence, displayed on the part of a stranger, is enough to make a fellow agree to live with his mother-in-law! We therefore willingly publish Mr. Shaw's advertisement and letter, free—he needn't pay us a cent. Everybody should at once order his medicine, if for no other reason than its cheapness. We have a great notion to try a ton or so of the pills, just to gain that "33 pounds of flesh in three months." We need it—need it terribly, for we have been sitting around in nothing but skin and bones, for the past thirty-two years or so. If we *should* try the experiment, we will let our readers know, and send them a photograph, so that they may know how we appear in our extra 38 pounds of flesh. Until then, we advise them to let Mr. Shaw and his free-formula-advertising friends alone.

---

LIMA, N. Y., May 27, 1871.

DR. UPDEGRAFF:

*Dear Sir:*—Do you know anything about "Cornea Restorers?" Would you advise me to try them instead of spectacles?

M. J. R.

Yes, if you desire to dislocate your lens, and have an operation for *cataract* shortly. Otherwise not.

---

SYRACUSE, N. Y., May 25, 1871.

THAD S. UPDEGRAFF, M. D.

*Dear Sir:*—Can you tell me where I can find a good Cancer Doctor, one that I can rely upon?

Mrs. M——.

Yes. Wherever you can find a skillful surgeon with a sharp knife. Better *keep* your cancer than go to these so-called "Cancer Doctors," who thrive by applying their "cancer plasters" to all sorts of tumors, and robbing you of your money and your life. Your cancer is the best thing of the two—hold on to it.

PENN YAN, N. Y., May 1, 1871.

DR. UPDEGRAFF:

*Sir:*—I bought a pair of spectacles of your agent who called at my house last week. He said that I could exchange them any time I chose if they did not suit me. If I return them will you send me a stronger pair.

L. R.

I guess not! There are several slight inaccuracies in your statement. First, our agent did not call at your house. Second, we have no agent. Third, we don't deal in spectacles; and finally, you are an infernal fool for buying spectacles of *any one* who peddles them from door to door.

---

WATERLOO, N. Y., April 30, 1871.

DR. UP DE GRAFF:

*Sir*—Hearing of your skill as a surgeon (just so—interjected by the editor), I write you to inquire what I had better do about my breast. I put a cancer plaster on it, recommended by Dr. ——, a very skillful cancer doctor, and now it is so sore, and all broken out in big, angry looking sore spots, and is so painful I do not know what to do. Will you please tell me.

MRS. ——.

Hang yourself. That is the next best treatment after patronizing a cancer doctor. We are sorry for you, but the above is the only *cure* we can prescribe for your difficulty. There was never a genuine cancer of the breast cured by means of a "cancer plaster," and we have known of hundreds of patients that have been hastened to a terrible death by such applications. The only cure for cancer of the breast is early amputation, at the hands of a regular surgeon.

---

NOTE.—Several communications received prior to April 11th, were destroyed by the fire which burned our place of business. They cannot therefore, be answered.—EDITOR.

---

PRESERVED MEAT.—Dr. Stein, of Dresden, while lecturing lately on the preservation of food, opened a tin canister of meat, preserved by what is known as Apert's method, and prepared by him in 1851. The meat, on examination, it is said, was found to be as fresh, and of as good a flavor, as when placed in the canister nineteen years previously.

---

OTHER JOURNALS.—See the list of journals with which we club. Send the publisher's price of any on the list to us, and you will receive it and the BISTOURY free.

